# Procedures of User-Centered Usability Assessment for Digital Solutions: Scoping Review of Reviews Reporting on Digital Solutions Relevant for Older Adults

**DOI:** 10.2196/22774

**Published:** 2021-01-13

**Authors:** Anabela G Silva, Hilma Caravau, Ana Martins, Ana Margarida Pisco Almeida, Telmo Silva, Óscar Ribeiro, Gonçalo Santinha, Nelson P Rocha

**Affiliations:** 1 School of Health Sciences Center for Health Technology and Services Research University of Aveiro Aveiro Portugal; 2 Department of Medical Sciences Institute of Electronics and Informatics Engineering of Aveiro Aveiro Portugal; 3 DigiMedia, Department of Communication and Art University of Aveiro Aveiro Portugal; 4 Department of Education and Psychology Center for Health Technology and Services Research University of Aveiro Aveiro Portugal; 5 Department of Social, Political and Territorial Sciences Governance, Competitiveness and Public Policies University of Aveiro Aveiro Portugal

**Keywords:** mobile phone, user-centered design, aged, review, telemedicine

## Abstract

**Background:**

The assessment of usability is a complex process that involves several steps and procedures. It is important to standardize the evaluation and reporting of usability procedures across studies to guide researchers, facilitate comparisons across studies, and promote high-quality usability studies. The first step to standardizing is to have an overview of how usability study procedures are reported across the literature.

**Objective:**

This scoping review of reviews aims to synthesize the procedures reported for the different steps of the process of conducting a user-centered usability assessment of digital solutions relevant for older adults and to identify potential gaps in the present reporting of procedures. The secondary aim is to identify any principles or frameworks guiding this assessment in view of a standardized approach.

**Methods:**

This is a scoping review of reviews. A 5-stage scoping review methodology was used to identify and describe relevant literature published between 2009 and 2020 as follows: identify the research question, identify relevant studies, select studies for review, chart data from selected literature, and summarize and report results. The research was conducted on 5 electronic databases: PubMed, ACM Digital Library, IEEE, Scopus, and Web of Science. Reviews that met the inclusion criteria (reporting on user-centered usability evaluation procedures for any digital solution that could be relevant for older adults and were published in English) were identified, and data were extracted for further analysis regarding study evaluators, study participants, methods and techniques, tasks, and test environment.

**Results:**

A total of 3958 articles were identified. After a detailed screening, 20 reviews matched the eligibility criteria. The characteristics of the study evaluators and participants and task procedures were only briefly and differently reported. The methods and techniques used for the assessment of usability are the topics that were most commonly and comprehensively reported in the reviews, whereas the test environment was seldom and poorly characterized.

**Conclusions:**

A lack of a detailed description of several steps of the process of assessing usability and no evidence on good practices of performing it suggests that there is a need for a consensus framework on the assessment of user-centered usability evaluation. Such a consensus would inform researchers and allow standardization of procedures, which are likely to result in improved study quality and reporting, increased sensitivity of the usability assessment, and improved comparability across studies and digital solutions. Our findings also highlight the need to investigate whether different ways of assessing usability are more sensitive than others. These findings need to be considered in light of review limitations.

## Introduction

### Background

Digital solutions, defined as any set of technologies, systems, and mobile apps that are available on a digital device such as an iPad, a laptop, or a smartphone [1], have become popular in different areas, namely to optimize and personalize health care provision [2], to promote healthy lifestyles (eg, physical activity) [3,4], to minimize loneliness and social exclusion by promoting social, religious, civic, and political participation [5-7], or to improve safety, independence, and confidence [2].

The accelerated aging of the population imposes several challenges on the health care and social systems. Owing to the higher rates of disease and morbidity [8,9], digital solutions have been noted as a valid contributor to help reach a high number of individuals at lower costs [10]. However, developing digital solutions adjusted to older adults presents specific challenges related to age and disease, such as loss of visual and hearing acuity or changes in fine motricity. These need to be considered so that the technology matches the users’ needs and characteristics and, ultimately, its use results in an added value in daily life [11,12]. To guarantee that a digital solution is fully adjusted to its users, a robust evaluation process must be considered [13]. One of the key attributes of digital solutions that require careful attention and evaluation is usability.

Usability is part of the user experience, that is, the total usage phenomenon [14], and is defined as the measure by which a product can be used by specific users to achieve specific goals with effectiveness, efficiency, and satisfaction in a specific context of use [15]. Efficacy refers to the degree of accuracy and completeness with which users achieve certain goals in a given environment, efficiency is related to the accuracy and completeness of the goals achieved with regard to the resources used, and satisfaction is defined as the comfort and acceptance on the use of a system [15]. Furthermore, the level of usability obtained depends on the specific circumstances in which the product is used and the usage context includes users, tasks, equipment (hardware and software), and the physical and social environment, as all of these factors can influence the usability of digital solutions [15]. In other words, usability is the ability of a product to be understood, learned, used, and attractive to the user, when used under specific conditions. This definition reinforces the idea that a product has no intrinsic usability and only the ability to be used under specific conditions [16]. Good usability allows reducing task execution times, errors, or learning times; improves user satisfaction; and leads to improved product acceptability, increased user satisfaction, and improved product reliability [17].

Usability evaluation is an important part of the overall development of user interaction mechanisms, which consists of interactive cycles of design, prototyping, and validation [18]. Ideally, usability evaluation must be present at all development stages and must be iterative to enable a continuous evolution of the quality of the product or service. The literature describes several models, methods, and techniques to ensure that usability issues are considered during the development process. The selection of these models, methods, and techniques depends on the development stage of digital solutions and available resources [19]. Certain models of usability assessment rely on usability experts, whereas others rely on end users (user-centered usability assessment). The former are known as the analytical models [20] and involve the inspection of the digital solution by experts to assess the various aspects of user interaction against an established set of principles of interface design and usability [21,22]. The latter refer to the empirical models [20] and involve having the perspective of users and are key to highly usable digital solutions by ensuring that the digital solutions meet the users’ needs and requirements, that is, they are adapted to the body and mind of their user in a given context [23]. This perspective is gathered using different methods (eg, test and inquiry) and techniques (eg, interviews, think-aloud, and observation), which are usually combined [24]. Both models are essential in the development process of digital solutions and provide complementary information [25]. This review focuses on the users’ assessment of usability.

Usability assessment involving users is a complex task, and the use of only one method (eg, test or inquiry) may not be comprehensive enough to thoroughly consider all relevant issues associated with a given product or service [19]. In addition, different methods have different strengths and weaknesses and provide information on different aspects of the digital solution [19]. Nevertheless, it is important to standardize the evaluation and reporting of usability procedures across studies. This will guide researchers, facilitate comparisons across studies, promote high-quality usability studies, which would be more likely to identify usability problems, and provide relevant data that contribute to highly usable solutions. The first step to standardizing is to provide an overview of how user-centered usability evaluation procedures are reported in the literature.

### Objective

This scoping review of reviews aims to synthesize the procedures used or reported for the different steps of the process of conducting a user-centered usability assessment of digital solutions relevant for older adults and identify potential gaps in the present reporting of procedures. The secondary aim is to identify the principles guiding this assessment.

## Methods

### Study Design

This study followed the 5-stage scoping review methodology defined by Levac et al [26] based on the framework previously developed by Arskey and O’Malley [27]. The stages include (1) identification of the research question, (2) identification of relevant studies, (3) selection of relevant studies, (4) charting the data, and (5) collating, summarizing, and reporting the results of the review. A scoping review of the literature aims to map key concepts, summarize a range of evidence, especially in complex fields, and identify gaps in the existing literature. It allows for broader perspectives in comparison with systematic reviews [26,27] and, therefore, was the appropriate approach for this study, in which we aimed to cover a broad range of usability evaluation procedures and identify gaps to direct future research.

### Identification of the Research Question

The research question provides a roadmap for the subsequent stages of the review. It was defined based on the analysis of the literature in the field of usability evaluation of digital solutions and the expertise of the research team, that is, during our previous work in the field of usability evaluation, we identified a lack of consensus in the academic literature regarding the instruments, protocols, and methodologies used for assessing usability across a range of digital solutions (eg, websites, assistive technology, augmented reality). Therefore, to have a more in-depth knowledge of the practices and procedures used, the following research question was defined: *What are the current practices for the user-centered assessment of the usability of digital solutions (eg, procedures instruments) relevant (ie, that could be used and have value) for the older adult population?* This broad question was subdivided into 5 research questions: (1) What are the characteristics of study evaluators reported in user-centered usability studies for digital solutions relevant to older adults? (2) What are the characteristics of study participants reported in user-centered usability studies for digital solutions relevant to older adults? (3) How are the tasks used for user-centered usability studies for digital solutions relevant to older adults? (4) What are the methods and techniques used in user-centered usability studies for digital solutions relevant to older adults? and (5) Where (ie, the environment) do user-centered usability evaluations take place?

### Identification of Relevant Studies

The search expression *usability* OR *user experience* was used in the electronic search carried out in PubMed, ACM Digital Library, IEEE, Scopus, and Web of Science. The search expression did not include *older adults* as we did not want to limit the inclusion of reviews to those specifically mentioning *older adults*. Databases were searched for English language reviews published between January 1, 2009, and January 23, 2020. The limit of 2009 was established, as 2007 was the year the *ambient assisted living* joint programme was launched by the European Commission, which is a transnational funding program exclusively focused on the research and development of digital solutions directed at older adults [28]. Therefore, we searched for reviews from 2009 onward that covered the primary studies published after 2007.

### Selection of Relevant Studies

All references were imported into Mendeley software (Elsevier, North-Holland) through which duplicates were removed. The first 300 abstracts were screened by 3 reviewers (HC, AS, and NR). Differences in judgment were used to refine the inclusion and exclusion criteria and were discussed until consensus was reached. This first phase of screening also served to build a common understanding of the inclusion and exclusion criteria. Screening of the remaining abstracts was performed by 1 reviewer (HC). Similarly, the first 10 full articles were screened by 2 reviewers (HC and AS), and differences in judgment were discussed until consensus was reached. If consensus was difficult to attain, a third reviewer who is a senior reviewer and an expert on usability (NR) was consulted. The remaining full papers were independently screened by one of these 3 reviewers.

To be included in this scoping review, studies had to report on user-centered usability procedures or methods of evaluation for any type of digital solution that could be relevant for older adults and that was (1) published in English; (2) a review, either systematic, scoping, or narrative review; (3) addressing and synthesizing evidence on any of the steps or methodologies used for usability assessment; and (4) addressing usability in general or for a specific digital solution that was considered relevant (this was a subjective judgment made by the authors of the review) to older adults or those caring for older adults, such as informal caregivers, family members, or health care professionals.

Studies were excluded if they (1) were grossly unrelated to the study topic (eg, chemistry field); (2) targeted children or younger age groups (eg, digital solutions for children with diabetes); (3) addressed usability for nondigital solutions (eg, buildings) or digital solutions assessed as not of interest for older adults or those caring for them (eg, moodle and eLearning solutions); and (4) addressed usability of digital solutions for caregivers of older adults, but only those studies that did not involve interaction or feedback with older persons or those caring for them were included.

### Charting the Data and Collating, Summarizing, and Reporting the Results

The data extraction tool was developed using an iterative team process. The preliminary data extraction categories were derived from our research questions. The following data were extracted from each review: authors, year of publication, purpose/aim of the study, and the number of studies included in the review. Further extraction, analysis, and reporting of results were guided by the framework proposed by Ellsworth et al [29] for reporting usability evaluations, and the following operational definitions were used for this review:

1. Study evaluators, that is, the individuals who conducted the usability evaluation.
2. Participants, that is, the individuals who were asked to evaluate the usability of a product or service.
3. Tasks, that is, the activities that participants were asked to perform when evaluating the usability of a product or service.
4. Methods and techniques: methods refer to the set of techniques used to perform formative user-centered usability evaluation of a certain type at any stage of the product or service development. Usability evaluation techniques refer to a set of procedures used to perform a usability evaluation and collect data of a certain type. For this review, methods and techniques of usability evaluation were categorized and defined as presented in Table 1 (adapted from Martins et al [30]). Usability assessment usually requires the combination of more than one method, can be conducted remotely (ie, evaluators are separated in space from users) or in the presence of the participants, and can be synchronous (ie, occur at the time of the participants’ interaction with the system) or asynchronous [30].
5. The test environment, that is, the environment where the evaluation of usability takes place: (1) laboratory or controlled conditions, usually a transversal assessment, or (2) in a real context, that is, the usability assessment is carried out in the same context and circumstances where the end product or service is expected to be used, which is usually a longitudinal assessment.

Details on the characteristics of each of these components of the usability assessment were extracted.

**Table 1:** Methods of user-centered usability evaluation.

Method and definition and technique for data collection		Definition
**Test: involves observing users while they perform predefined tasks and consists of collecting mostly quantitative data; the test is centered on the interaction of the user with the technology**		
	Performance evaluation	Evaluated by recording elements related to the execution of a particular task (eg, execution time, success or failure, number of errors, eye-tracking, and automated usability evaluation or logfiles or web usage analysis or app-use generated data or sensor data)
	Observation	Attentive visualization and systematic recording of a particular phenomenon, including people, artifacts, environments, behaviors, and interactions. Observation can be direct, when the researcher is present during the task execution, or indirect, when the task is observed through other means such as video recording
	Think-aloud	Users are invited to talk about what they see, do, think, or feel as they interact with the system or service
**Inquiry: provide valuable, subjective, and usually qualitative information on the users’ opinions and expectations**		
	Focus groups	Involves a small number of people in an informal discussion
	Interviews	Involves a one-to-one interaction to gather opinions, attitudes, perceptions, and experiences
	Scales/questionnaires	Collects data on characteristics, thoughts, feelings, perceptions, behaviors, or attitudes, measuring either one (scale) or several (questionnaire) dimensions of usability. It is important to distinguish whether instruments were validated
	Diary studies	Users record events related to their experience in the context of daily activity and later share them with the evaluators
	Card sorting	It involves participants using logic while sorting content or *cards* into categories or groups that make sense to them, given the information they are provided with

## Results

### Overview

**Figure 1:**
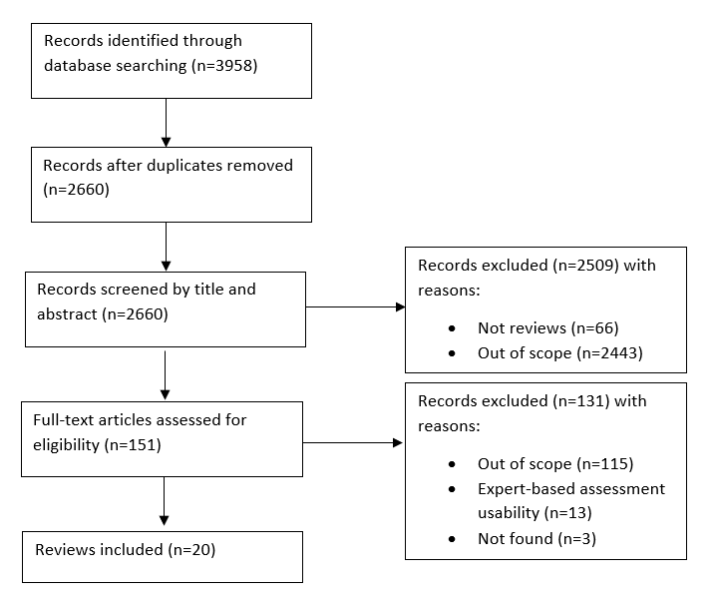
Flow diagram showing study identification and selection for the present review.

The PRISMA (Preferred Reporting Items for Systematic Reviews and Meta-Analyses) flow diagram for this scoping review is presented in Figure 1. A total of 3958 articles were identified from the 5 electronic databases. Of these, 1298 were eliminated because they were duplicates or did not have the author’s name. The remaining 2660 records were screened based on title and abstract and 2509 were excluded because they were not reviews (66/2660, 2.48%) or were out of scope (2443/2660, 91.8%). A total of 151 full texts were read for further analysis. Of these, 115 manuscripts were excluded because they were not related to usability, 3 articles were not found, and 13 reported on the assessment of usability by experts. Therefore, 20 reviews were included in this scoping review of the reviews. Of these, 19 were systematic reviews and one was a narrative review. Table 2 presents the main characteristics of the included reviews (study, purpose, and number of included studies).

**Table 2:** General characteristics of included reviews.

Study	Purpose of the review	Number of studies included in the review
Ellsworth et al (2017) [29]	Review methods employed for usability testing on electronic health records	120
Allison et al (2019) [31]	Review methodologies and techniques to evaluate websites; provide a framework of the appropriate website attributes that could be applied to any future website evaluations	69
Azad-Khaneghah et al (2020) [32]	Review the rating scales used to evaluate usability and quality of mobile health applications	87
Baharuddin et al (2013) [33]	Propose a set of usability dimensions that should be considered for designing and evaluating mobile applications	Not referred
Bastien (2010) [34]	List test procedures and define and develop tools to help conduct user tests	Not referred (narrative review)
Bhutkar et al (2013) [35]	List the most commonly applied usability evaluation methods and related emerging trends	30
Cavalcanti et al (2018) [36]	Understand which methods and user assessment approaches are commonly used in motor rehabilitation studies that use augmented reality applications	32
Fernandez et al (2012) [37]	Analyze the usability evaluation methods that have proven to be the most effective in the web domain	18
Fernandez et al (2011) [38]	Analyze the usability evaluation methods that have been employed to evaluate web applications over the last 14 years	206
Fu et al (2017) [39]	Assess the usability of diabetes mobile apps developed for adults with type 2 diabetes	7
Hussain et al (2014) [40]	Review the relevant and appropriate usability dimensions and measurements for banking applications	49
Inal et al (2020) [41]	Analyze how usability is being addressed and measured in mobile health interventions for mental health problems	42
Klaassen et al (2016) [42]	Analyze if usability methods are equally employed for different end-user groups and applications	127
Lim et al (2019) [43]	Identify, study, and analyze existing usability metrics, methods, techniques, and areas in mobile augmented reality learning	72
Narasimha et al (2017) [44]	Analyzing the characteristics of usability-related studies conducted using geriatric participants and the subsequent usability challenges identified	16
Shah and Chiew (2019) [45]	Identify, analyze, and synthesize the usability features and assessment approaches of pain management mobile applications targeted at the evaluation studies	27
Simor et al (2016) [46]	Analyze usability evaluation methods used for gesture-based games, considering devices with the motion-sensing capability	10
Sousa and Lopez (2017) [47]	Identify psychometrically tested questionnaires that measure the usability of eHealth tools	35
Yen and Bakken (2012) [48]	Review and categorize health information technology usability study methods, and to provide practical guidance on health information technology usability evaluation	346
Zapata et al (2015) [49]	Review a set of selected papers that perform a usability evaluation of mobile health–related mobile apps	22

### Study Evaluators

Only 4 out of the 20 (20%) [29,36,37,46] included reviews briefly mentioned any characteristic of the evaluators' profile. One of the reviews [36] reported that one of the 32 articles included mentioned that the person who performed the usability assessment was a blind evaluator. One review stated that several studies (exact numbers not provided) used graduate students as both evaluators to perform usability inspections and participants in experimental sessions (eg, think-aloud protocol, remote user testing) [37], whereas another review [46] reported that usability evaluations were conducted by researchers. In a review by Ellsworth et al [29], 29% (35/120) of the included articles presented the description of the study evaluators responsible for designing and carrying out the usability evaluation, but the characteristics reported in primary studies were not provided.

### Participants

Half of the reviews included in this scoping review did not refer to the characteristics of the participants included in the primary studies reviewed. Of the reviews, 50% (10/20) reviews that reported on any of the participants' characteristics, 4 reported mean age or age range [36,41,46,49], 4 reported the gender of participants [36,41,44,46], 8 reported the sample size [35,36,39,41,42,46,47,49], and 7 reported on other characteristics of participants by describing them as healthy participants or as having a specific clinical condition [36,37,39,41,44,46,49]. Nevertheless, 20% (4/20) reviews that reported the age of the participants also reported that not all primary studies detailed such information. Similar findings were reported for gender and sample size. No reference to sample size calculation or rationale for deciding on sample size was provided. Other characteristics of participants mentioned were being healthy, having a specific clinical condition, belonging to a specific occupational group (health care providers or students), and previous experience with mobile devices. Multimedia Appendix 1 presents a description of the information provided within the included reviews.

### Tasks

Only 2 of the 20 (10%) included reviews referred to the tasks that participants were asked to perform for the usability evaluation [46,49]. Simor et al [46] conducted a usability evaluation for gesture-based games and reported that the games and, consequently, the usability evaluation of each study had different aims, target populations, interfaces, and details, but in the majority of the studies, the protocol used was presented. Zapata et al [49] performed a systematic review on mobile health apps and reported that 17 of the 22 primary studies included reported the number of tasks performed by the users. The number of tasks ranged between 1 and 25.

### Methods and Techniques

Of the 20 systematic reviews included, only 3 (15%) [33,40,41] did not refer to the methods and techniques of usability used. Among the inquiry methods, the questionnaires/scales (15/20, 75%) and interviews (12/20, 60%) were most commonly reported. Among the test methods, the techniques of performance (9/20, 45%) and think-aloud were the most commonly reported (6/20, 30%; Table 3). Of the 20 reviews, 6 (30%) reported on combinations of techniques mentioning a total of 22 different combinations of 4, 3, or 2 techniques. Most combinations include at least one technique from each method, which indicates that a multimethod approach was used (Table 4). Among scales/questionnaires, which constitute the technique most often reported, the most common usability assessment scales were the System Usability Scale [29,32,41-43,46,47] and the Post-Study System Usability Questionnaire [41,42,46,47]. The other scales/questionnaires include the Questionnaire for User Interaction Satisfaction [29,42,47], the Software Usability Measurement Inventory [32,42], the Usefulness, Satisfaction, and Ease of use Questionnaire [32,41], the Computer System Usability Questionnaire [32,47], the After-Scenario Questionnaire [46,47], the Perceived Useful and Ease of Use [32], the IsoMetrics usability inventory [32], the Health Information Technology Usability Evaluation Scale [32], the user Mobile Application Rating Scale [32]; the IBM ease of use [42], and the ISO 9241–11 Questionnaire [43]. In addition, several reviews have reported the use of nonvalidated questionnaires [32,41,43,46]. One review reported that 26% of the included studies used a remote assessment of usability, where participants are in an uncontrolled environment [31].

**Table 3:** Detailed techniques used for usability evaluation.

Study	Test			Inquiry				
	Performance evaluation (n=9)	Observation (n=3)	Think-aloud (n=6)	Focus group (n=3)	Interview (n=12)	Scales or questionnaires (n=15)	Diary studies (n=1)	Card sorting (n=1)
Allison et al (2019) [31]	✓^a^	—^b^	—	—	—	✓	—	—
Azad-Khaneghah et al (2020) [32]	—	—	—	—	—	✓	—	—
Bastien (2010) [34]	—	—	—	—	✓	—	✓	—
Bhutkar et al (2013) [35]	✓	—	✓	—	✓	—	—	—
Cavalcanti et al (2018) [36]	✓	—	✓	—	—	✓	—	—
Ellsworth et al (2017) [29]	—	—	—	✓	✓	✓	—	✓
Fernandez et al (2012) [37]	✓	—	✓	—	✓	✓	—	—
Fernandez et al (2011) [38]	✓	—	✓	✓	✓	✓	—	—
Fu et al (2017) [39]	✓	—	—	—	—	✓	—	—
Klaassen et al (2016) [42]	✓	✓	—	—	✓	✓	—	—
Lim et al (2019) [43]	✓	—	—	—	✓	✓	—	—
Narasimha et al (2017) [44]	—	—	—	—	✓	✓	—	—
Shah and Chiew (2019) [45]	—	✓	—	—	✓	✓	—	—
Simor et al (2016) [46]	—	—	—	—	✓	✓	—	—
Sousa and Lopez (2017) [47]	—	—	—	—	—	✓	—	—
Yen and Bakken (2012) [48]	—	✓	✓	✓	✓	✓	—	—
Zapata et al (2015) [49]	✓	—	✓	—	✓	✓	—	—

^a^Reported in the review.

^b^Not reported.

**Table 4:** Detailed description of the combination of techniques used for usability assessment.

Techniques	Study						Multimethod
	Cavalcanti et al (2018) [36]	Fu et al (2017) [39]	Inal et al (2020) [41]	Shah & Chiew (2019) [45]	Simor et al (2016) [46]	Zapata et al (2015) [49]	
Observation + performance evaluation + think-aloud + scale/questionnaire	✓^a^	N/A^b^	N/A	N/A	N/A	N/A	✓
Observation + performance evaluation + scale/questionnaire + interview	✓	N/A	N/A	N/A	N/A	N/A	✓
Observation + scale/questionnaire+ interview + diary studies	N/A	N/A	✓	N/A	N/A	N/A	✓
Performance evaluation + think-aloud + scale/questionnaire + interview	N/A	N/A	✓	N/A	N/A	N/A	✓
Observation + performance evaluation + think-aloud + interview	N/A	N/A	✓	N/A	N/A	N/A	✓
Performance evaluation + scale/questionnaire + interview	✓	N/A	✓	✓	N/A	N/A	✓
Performance evaluation + scale/questionnaire + focus group	N/A	N/A	✓	N/A	N/A	N/A	✓
Performance evaluation + scale/questionnaire + observation	✓	N/A	✓	N/A	N/A	N/A	✓
Performance evaluation + observation	N/A	N/A	✓	N/A	✓	N/A	N/A
Think-aloud + scale/questionnaire + interview	N/A	N/A	✓	✓	N/A	N/A	✓
Think-aloud + scale/questionnaire + interview	N/A	✓	N/A	N/A	N/A	N/A	✓
Scale/questionnaire + interview + focus group	N/A	N/A	✓	✓	N/A	N/A	N/A
Observation + scale/questionnaire + interview	✓	N/A	✓	✓	N/A	N/A	✓
Observation + scale/questionnaire	✓	✓	✓	N/A	N/A	N/A	✓
Observation + interview	N/A	N/A	N/A	✓	N/A	N/A	✓
Performance evaluation + observation	✓	N/A	N/A	N/A	N/A	N/A	N/A
Performance evaluation + scale/questionnaire	✓	N/A	✓	N/A	N/A	✓	✓
Think-aloud + scale/questionnaire	N/A	N/A	✓	N/A	N/A	N/A	✓
Think-aloud +interview	N/A	N/A	N/A	✓	N/A	N/A	✓
Scale/questionnaire + interview	✓	N/A	✓	✓	N/A	✓	N/A
Scale/questionnaire + diary studies	N/A	N/A	✓	N/A	N/A	N/A	N/A
Interview + focus group	N/A	N/A	✓	N/A	N/A	N/A	N/A

^a^Reported in the review.

^b^N/A: not applicable.

### Test Environment

Of the 20 reviews, 2 (10%) reported on the environment where the usability assessment of the included studies took place. In a review by Bhutkar et al [35], of the 17 studies that reported on the test environment, 8 were conducted in hospitals, 5 in intensive care units, and 4 in laboratories. In addition, 31 of the 42 studies reviewed by Inal et al [41], which focused on mobile health interventions for mental health problems, reported having conducted their usability testing in the natural environment of the participants with the technology deployed in the everyday environment of the intended users or their representatives. In addition, the review of Ellsworth et al [29] did not provide data on the test environment; however, the test environment was an inclusion criterion, as they stated that they have included studies that tested the usability of the hospital and clinic electronic health records in the inpatient, outpatient, emergency department, or operating room settings.

## Discussion

### Principal Findings

This scoping review of reviews aims to synthesize the procedures used or reported for the different steps of the process of conducting a user-centered usability assessment of digital solutions relevant for older adults, to identify gaps in the literature, and to identify the best practices for each of the different steps. The results suggest that the characteristics of study evaluators and participants and task procedures are only briefly reported, and no agreement seems to exist on what should be reported. The methods and techniques used for the assessment of user-centered usability are the topics most commonly and comprehensively reported in the reviews, whereas the test environment is seldom and poorly characterized. Despite our aim of searching for reviews reporting on digital solutions relevant for older adults, only one of the included reviews specifically targeted older adults. This suggests that studies using older adults are scarce and that the findings of this scoping review also apply to usability studies with adults.

Our findings are in line with the review of Ellsworth et al [29], who reported that several of the included studies described the participants, but not the individual who conducted the usability assessment (study evaluator). The level of expertise and domain experience, whether the study evaluator is external to the team developing the product or service being assessed or, on the contrary, is part of the team and potentially has a conflict of interest when assessing usability, are examples of aspects that have the potential to influence the results of the usability assessment. Therefore, these should be reported by the authors. Most of the techniques are complex procedures of usability assessment; some of these depend on the interaction between the participant and the study evaluator and, therefore, require experience and knowledge to be assessed effectively.

The characteristics of the study participants most commonly reported across reviews were age and sex. However, these seem insufficient for the reader to make a judgment regarding the degree of similarity between the sample and the target end users. Educational or digital literacy levels are likely to influence how the participant perceives the usability of the system. For example, different subgroups of older adults may perceive the usability of the same system differently [46]. Therefore, a detailed characterization of physical, emotional, cognitive, and digital skills is needed for an appropriate interpretation of the results of the usability evaluation in certain subgroups of older adults. Furthermore, a detailed characterization of health conditions might also be relevant [46]. These aspects will also inform whether the sample used is representative of the end users. The use of nonrepresentative users and, therefore, the failure to consider their needs and preferences may result in products with low usability [36]. In general, the sample sizes are small, and no rationale for the size of the sample is provided. The appropriate sample size for usability studies is a matter of debate, with some authors arguing that 4 or 5 participants are enough to identify approximately 80%-85% of usability problems [50-52], whereas others report that with these numbers of participants only 35% of usability problems are determined [53]. The type of interfaces, the tasks performed by the participants, the context of use, and the state of technology development may explain the differences between studies [34]. Furthermore, it is worth noting the definition of usability as the measure by which a product can be used by specific users to achieve specific goals with effectiveness, efficiency, and satisfaction [15]. Conceivably, small sample sizes may be enough to detect usability problems but may be insufficient to have a broader view of usability more in line with the present definition.

Only 2 reviews reported on the tasks that participants were asked to perform to assess the usability of the product or service [46,49], and both concluded that, in general, studies reported on the protocol of the tasks used. Tasks vary depending on several factors, such as study aims, target population, interfaces, methods, and techniques used for usability assessment [46]. Nevertheless, the definition or selection of tasks that participants should perform should mirror the future use of the product or service [34,40]. No principles were found to guide the selection of tasks. For example, should there be a minimum set of tasks to be performed, should tasks require single or multiple steps, or should there be a minimum amount of time that each participant needs to spend using the product or service are illustrative examples of issues that are not clear.

The methods and techniques used for the assessment of usability have been consistently reported, and most reviews have found that a combination of methods and/or techniques are usually performed, in line with recommendations [19]. Different methods and techniques have different strengths and limitations [46] and, therefore, their combination is more likely to provide a comprehensive view of usability problems [19]. For example, scales and questionnaires are easy to use and useful for gathering self-reported data about the user’s perception but might have limited value informing on which aspects of the system need to be targeted for improvement [29,54]. Scales and questionnaires should be valid, but a few reviews have reported the use of scales and questionnaires that are unlikely to have been validated. Although there might be reasons to develop or adapt a scale/questionnaire, this process must be followed by evidence of its validity [41]. Interviews and observations are recommended when the number of participants is small because both generate high amounts of data that are time-consuming to analyze. Nevertheless, interviews can be useful to understand the reasoning of the user when facing a problem, and observation gives an insight into the moment when a problem occurs [46]. It is argued that think-aloud protocols may result in the loss of focus on the tasks being performed, whereas user performance is an easy assessment, particularly in cases where the system automatically records the performance indicators, but might provide limited information if used alone [46]. The most frequent multimethod combination described in the literature is the test and inquiry method combination; however, we found no information in the included reviews regarding which combination of techniques is the most sensitive and whether this could vary depending on the development stage of the product or service being evaluated. Furthermore, the combination of techniques should allow for the assessment of effectiveness, efficiency, and satisfaction, as these are all part of usability.

Only 2 reviews reported on the test environment, but both referred that most included studies reported usability testing to have been conducted in the real context. Nevertheless, we found no indication of how long the usability assessment should be conducted, that is, how long the participants should be allowed to use the product or service before assessing it, and whether conducting the usability assessment in a real context means that the product or service was used in the circumstances that it is expected to be used.

### Recommendations and Future Research

The conducting of rigorous experiments on user-centered usability is likely to result in increased sensitivity for these experiments, that is, an increased ability to detect usability issues. Developing a consensus framework is likely to improve the quality of studies on usability evaluation and respective reporting, improve comparability of usability results across studies, provide digital solutions helping consumers and producers to identify the best products, improve the efficiency of the process of usability evaluation and facilitate further research on the impact of usability on other outcomes, such health-related outcomes. Textbox 1 presents a list of parameters that we believe should be considered when planning and reporting user-centered usability studies. These parameters provide guidance while also being flexible to accommodate study differences regarding aspects such as study participants or the digital solution being assessed. At present, we are working on a Delphi-study aiming to establish an international consensus on user-centered usability evaluation procedures.

**A proposed guide of aspects to consider when designing and reporting a user-centered usability evaluation study.**

Study evaluator:

- Provide a rationale for sample size
- Experience with usability evaluation with users (if none, plan training)
- Establish clear inclusion and exclusion criteria (age, gender, educational level, and academic background)
- Clarify whether internal or external to product development

Participants:

- Provide a rationale for sample size
- Define clear inclusion and exclusion criteria
- Define sampling methods (probability/nonprobability) and setting of recruitment

Methods and techniques:

- Provide a rationale for the combination of methods and techniques
- Define equipment needed
- Select valid and reliable instruments of assessment

Task:

- Define the number
- Provide a detailed description of tasks
- Develop a participant script

Test environment/equipment:

- Identify and justify the choice (lab test or field test or both; remote test or face to face)
- Identify facilities and material needed
- Ensure the existence of an observation room and recording room
- Ensure the proper functioning of all equipment necessary for the test evaluation

### Limitations of This Scoping Review

Some limitations are directly related to the typology of this review, such as the absence of assessment of the quality of the included reviews and the quantitative summary of findings [55]. Usability is also a topic on which a large number of publications are published as conference proceedings, and such publications were not specifically searched (selection bias). Nevertheless, it is likely that by including mostly reviews published in journals that these are more comprehensive, as conference proceedings tend to have lower word counts for included papers. Abstracts and full-text screening were performed first by 3 and 2 authors, respectively, and after a common understanding was built, only 1 reviewer screened the remaining abstracts and full papers. Although we believe that this did not have a major impact on the results, having only 1 person screening for inclusion might have increased the possibility of error and of not including a potentially relevant study. The judgment made to decide whether a manuscript was on a product or technology that could be of use for older adults was a subjective judgment made by the authors and could have biased the results toward the field of health. Finally, no cross-checking of the primary studies included in each review was made and, therefore, the same primary studies could have been included in more than one review.

In summary, we found a lack of a detailed description of several steps of the process of assessing the usability of digital solutions and no evidence on good practices. These findings suggest the need for a consensus framework on the assessment of usability that informs researchers and allows standardization of procedures. Furthermore, it highlights the need to investigate whether different techniques of assessing usability are more sensitive than others to detect usability issues.

## Appendix

Multimedia Appendix 1 Summary details of participant profile and sample size (sometimes percentages do not add up to 100%, as only partial information was provided in the review).

## References

[1] Beschorner B, Woodward L, Karchmer-Klein R, Pytash KE (2020). Engaging teachers in a digital learner-centered approach to support understanding foundational literacy. Effective Practices in Online Teacher Preparation for Literacy Educators.

[2] Wagner F, Basran J, Bello-Haas VD (2012). A review of monitoring technology for use with older adults. J Geriatr Phys Ther.

[3] Simões P, Silva AG, Amaral J, Queirós A, Rocha NP, Rodrigues M (2018). Features, behavioral change techniques, and quality of the most popular mobile apps to measure physical activity: systematic search in app stores. JMIR Mhealth Uhealth.

[4] Silva AG, Simões P, Queirós A, Rodrigues M, Rocha NP (2020). Mobile apps to quantify aspects of physical activity: a systematic review on its reliability and validity. J Med Syst.

[5] Gilson A, Dodds D, Kaur A, Potteiger M, Ii JH (2019). Using computer tablets to improve moods for older adults with dementia and interactions with their caregivers: pilot intervention study. JMIR Form Res.

[6] Leone C, Lim JS, Stern A, Charles J, Black S, Baecker R (2018). Communication technology adoption among older adult veterans: the interplay of social and cognitive factors. Aging Ment Health.

[7] Rogers WA, Mitzner TL (2017). Envisioning the future for older adults: autonomy, health, well-being, and social connectedness with technology support. Futures.

[8] Fortin M, Hudon C, Haggerty J, van den Akker M, Almirall J (2010). Prevalence estimates of multimorbidity: a comparative study of two sources. BMC Health Serv Res.

[9] Fortin M, Bravo G, Hudon C, Vanasse A, Lapointe L (2005). Prevalence of multimorbidity among adults seen in family practice. Ann Fam Med.

[10] de la Torre-Díez I, López-Coronado M, Vaca C, Aguado JS, de Castro C (2015). Cost-utility and cost-effectiveness studies of telemedicine, electronic, and mobile health systems in the literature: a systematic review. Telemed J E Health.

[11] Hajek A, Jens-Oliver B, Kai-Uwe S, Matschinger H, Brenner H, Holleczek B, Haefeli WE, Heider D, Hans-Helmut K (2018). Frailty and healthcare costs-longitudinal results of a prospective cohort study. Age Ageing.

[12] Cheung JT, Yu R, Wu Z, Wong SY, Woo J (2018). Geriatric syndromes, multimorbidity, and disability overlap and increase healthcare use among older Chinese. BMC Geriatr.

[13] Herndon JH, Hwang R, Bozic KH (2007). Healthcare technology and technology assessment. Eur Spine J.

[14] Pyla PS, Hartson R (2019). Agile UX Design for a Quality User Experience.

[15] (2018). ISO 9241-11:2018(en) Ergonomics of Human-system Interaction — Part 11: Usability: Definitions and Concepts. ISO.

[16] Berntsen NO, Dybkjær L (2010). Multimodal Usability.

[17] Nunes IL (2006). Ergonomics and usability – key factors in knowledge society. Enterp Work Innov Stud.

[18] Martins Ai, Queirós A, Silva AG, Rocha NP, Saeed S, Bajwa IS, Mahmood Z (2014). Usability evaluation methods: a systematic review. Human Factors in Software Development and Design.

[19] Morrissey K (2014). A review of 'universal methods of design: 100 ways to research complex problems, develop innovative ideas, and design effective solutions'. Visitor Studies.

[20] Dix A, Finlay G, Abowd G, Beale R (2004). Human-Computer Interaction.

[21] da Costa RP, Canedo ED, de Sousa RT, de Oliveira RA, Villalba LJ (2019). Set of usability heuristics for quality assessment of mobile applications on smartphones. IEEE Access.

[22] Dix A, Finlay G, Abowd G, Beale R (2004). Human-Computer Interaction.

[23] Bernsen N, Dybkjær L (2010). Multimodal Usability.

[24] Martins AI, Queirós A, Rocha NP, Santos BS (2013). Avaliação de usabilidade: uma revisão sistemática da literatura. Iber J Inf Syst Technol.

[25] Queirós A, Silva A, Alvarelhão J, Rocha NP, Teixeira A (2013). Usability, accessibility and ambient-assisted living: a systematic literature review. Univ Access Inf Soc.

[26] Levac D, Colquhoun H, O'Brien KK (2010). Scoping studies: advancing the methodology. Implement Sci.

[27] Arksey H, O'Malley L (2005). Scoping studies: towards a methodological framework. Int J Soc Res Methodol.

[28] (2007). Ambient Assisted Living. AAL Programme.

[29] Ellsworth MA, Dziadzko M, O'Horo JC, Farrell AM, Zhang J, Herasevich V (2017). An appraisal of published usability evaluations of electronic health records via systematic review. J Am Med Inform Assoc.

[30] Martins AI, Queirós A, Silva AG, Rocha NP (2014). Usability Evaluation Methods: A Systematic Review, Human Factors.

[31] Allison R, Hayes C, McNulty CA, Young V (2019). A comprehensive framework to evaluate websites: literature review and development of goodweb. JMIR Form Res.

[32] Azad-Khaneghah P, Neubauer N, Cruz AM, Liu L (2020). Mobile health app usability and quality rating scales: a systematic review. Disabil Rehabil Assist Technol.

[33] Baharuddin R, Singh D, Razali R (2013). Usability dimensions for mobile applications-a review. Res J Appl Sci Eng Technol.

[34] Bastien JC (2010). Usability testing: a review of some methodological and technical aspects of the method. Int J Med Inform.

[35] Bhutkar G, Konkani A, Katre D, Ray GG (2013). A review: healthcare usability evaluation methods. Biomed Instrum Technol.

[36] Cavalcanti VC, Santana MI, Gama AE, Correia WF (2018). Usability assessments for augmented reality motor rehabilitation solutions: a systematic review. Int J Comput Games Technol.

[37] Fernandez A, Abrahão S, Insfran E (2012). A Systematic Review on the Effectiveness of Web Usability Evaluation Methods. 16th International Conference on Evaluation & Assessment in Software Engineering.

[38] Fernandez A, Insfran E, Abrahão S (2011). Usability evaluation methods for the web: a systematic mapping study. Inf Softw Technol.

[39] Fu H, McMahon SK, Gross CR, Adam TJ, Wyman JF (2017). Usability and clinical efficacy of diabetes mobile applications for adults with type 2 diabetes: a systematic review. Diabetes Res Clin Pract.

[40] Hussain A, Abubakar HI, Hashim B (2014). Evaluating Mobile Banking Application: Usability Dimensions and Measurements. 6th International Conference on Information Technology and Multimedia.

[41] Inal Y, Wake JD, Guribye F, Nordgreen T (2020). Usability evaluations of mobile mental health technologies: systematic review. J Med Internet Res.

[42] Klaassen B, van Beijnum BJ, Hermens HJ (2016). Usability in telemedicine systems-a literature survey. Int J Med Inform.

[43] Lim KC, Selamat A, Alias RA, Krejcar O, Fujita H (2019). Usability measures in mobile-based augmented reality learning applications: a systematic review. Appl Sci.

[44] Narasimha S, Madathil KC, Agnisarman S, Rogers H, Welch B, Ashok A, Nair A, McElligott J (2017). Designing telemedicine systems for geriatric patients: a review of the usability studies. Telemed J E Health.

[45] Shah U, Chiew T (2019). A systematic literature review of the design approach and usability evaluation of the pain management mobile applications. Symmetry.

[46] Simor FW, Brum MR, Schmidt JD, Rieder R, de Marchi AC (2016). Usability evaluation methods for gesture-based games: a systematic review. JMIR Serious Games.

[47] Sousa V, Lopez KD (2017). Towards usable E-health. A systematic review of usability questionnaires. Appl Clin Inform.

[48] Po-Yin Y, Bakken S (2012). Review of health information technology usability study methodologies. J Am Med Inform Assoc.

[49] Zapata BC, Fernández-Alemán JL, Idri A, Toval A (2015). Empirical studies on usability of mHealth apps: a systematic literature review. J Med Syst.

[50] Nielsen J, Landauer TK (1993). A Mathematical Model of the Finding of Usability Problems. Conference on Human Factors in Computing Systems.

[51] Virzi RA (2016). Streamlining the design process: running fewer subjects. Proc Hum Factors Soc Annu Meet.

[52] Virzi RA (2016). Refining the test phase of usability evaluation: how many subjects is enough?. Hum Factors.

[53] Spool J, Schroeder W (2001). Testing Web Sites: Five Users Is Nowhere Near Enough. Human Factors in Computing Systems.

[54] Martins AI, Queirós A, Silva AG, Rocha NP (2016). ICF Based Usability Scale: Evaluating Usability According to the Evaluators' Perspective About the Users' Performance. 7th International Conference on Software Development and Technologies for Enhancing Accessibility and Fighting Info-exclusion.

[55] Munn Z, Peters MD, Stern C, Tufanaru C, McArthur A, Aromataris E (2018). Systematic review or scoping review? Guidance for authors when choosing between a systematic or scoping review approach. BMC Med Res Methodol.

