# Supplementary material for: Procedures of User-Centered Usability Assessment for Digital Solutions: Scoping Review of Reviews Reporting on Digital Solutions Relevant for Older Adults
Source: JMIR Hum Factors. 2021 Jan 13;8(1):e22774. doi: 10.2196/22774 (PMC7840284; doi:10.2196/22774)
Supplement: Multimedia Appendix 1 [file humanfactors_v8i1e22774_app1.docx]

**Table 3.** Summary details of participant profile and sample size (sometimes percentages do not add up to 100%, as only partial information was provided in the review).

| Study | Age (years) | Gender | Profile | Sample size |
| --- | --- | --- | --- | --- |
| Bhutkar et al (2013) [35] | - Did not report | - Did not report | - Did not report | - Majority of the studies defined group tests with fewer than 20 participants - 3 studies counted with more than 20 participants - Remaining studies did not state the sample size |
| Cavalcanti et al (2018) [36] | Mean age of 39.89 years   - 14 studies did not report the participants’ ages - 2 reported within an age range (20-30 years and 27-35 years) - 3 articles reported the mean ages per group | - 37% of men - 23% of women - 40% unknown | - A mix of healthy users (59%), patients (34%), and studies that investigated both profiles (7%) - Inclusion of patients required the users who needed treatment or follow-up treatment for motor rehabilitation - 1 study used 2 subject groups (healthy subjects vs patients) | - In the 32 articles, there were a total of 806 participants (an average of 25.2 participants per study) |
| Fernandez et al (2012) [37] | - Did not report | - Did not report | - Most of the 18 empirical studies used students as participants to perform usability experimental sessions | - Did not report |
| Fu et al (2017) [39] | - Did not report | - Did not report | - All the usability studies with end users included patients with diabetes | - 1 study: 32 patients - 2 studies: 10 patients in each - 1 study: 5 patients |
| Inal et al (2020) [41] | - 8 studies reported the age range of the participants - 6 provided the mean age - 2 did not provide this information | - 4 studies had an equal gender distribution - 1 recruited the same number of males and females in stage 1, but all males in stage 2 | - Patients or patient families with parent-child dyads - Health care providers - Healthy users   Some studies performed the tests with one or more user type groups | - Total sample size at baseline, in 3 studies (regardless of the number of groups) ranged from 5 (n=2) to 3977 |
| Klaassen et al (2016) [42] | - Did not report | - Did not report | - Did not report | - Sample size ranged between 4 and 279 participants |
| Narasimha et al (2017) [44] | - Did not report | - Patient selection in 4 studies was based on age only, that is, participants had to be aged 65 years or older | - 12 studies had specific criteria for patient inclusion - Specific health conditions were also criteria for participant selection in various studies | - Did not report |
| Simor et al (2016) [46] | - 1 study did not state the age of participants - 1 study that referred included participants as *seniors* - 8 studies included persons with ages between 18 to 90 years (age ranges detailed in the article) | - There is an almost equal distribution of men and women in the studies | - The youngest study participants recruited were university students | - Samples vary from 7 to 24 participants - 1 study did not report the sample size |
| Sousa and Lopez (2017) [47] | - Did not report | - Did not report | - Did not report | - Most of the studies did not present the sample size - 5 studies had sample size of 5 to 10 participants - 3 studies had sample size below 5 participants |
| Zapata et al (2015) [49] | - 1 study for the elderly was tested with people aged 65 years or older | - Did not report | - 82% of the reviewed studies included real end users as patients, doctors, nurses, or caregivers - 4 studies were not evaluated with real users - 1 article did not mention anything about the users - 7 papers state that the users who perform the evaluation have some experience with mobile devices or even own a personal smartphone | - 91% of the papers described their sample size as ranging between 4 and 194 users |
